# Modulation of Splenic B Cell Subsets during Experimental *Leishmania donovani* Infection in BALB/c Mice

**DOI:** 10.3390/pathogens10070814

**Published:** 2021-06-29

**Authors:** Koushik Mondal, Shantanabha Das, Khudiram Naskar, Syamal Roy

**Affiliations:** 1 CSIR-Indian Institute of Chemical Biology, Kolkata 700032, India; drsyamalroy@yahoo.com (S.R.); knaskar08@gmail.com (K.N.); 2 Department of Ophthalmology, Hamilton Eye Institute, The University of Tennessee Health Science Center, Memphis, TN 38163, USA; 3 Department of Zoology, Diamond Harbour Women’s University, Sarisha, South 24 Parganas, West Bengal 743368, India; shantanabha2008@gmail.com

**Keywords:** visceral leishmaniasis, antimony resistance, cytokines, Transitional B cell, Follicular B cell, B1a B cell

## Abstract

Sodium antimonials are one of the major and common drugs used against visceral form leishmaniasis (VL). However, the development of drug resistance makes it difficult to manage this disease. Current work investigates the modulation of splenic B cells during experimental infection with antimony-sensitive and -resistant *Leishmania* *donovani* infection. Here we phenotypically characterized splenic B cell subsets in BALB/c mice infected with antimony drug-sensitive and -resistant VL strains using flow-cytometry method. In the splenocytes we noticed increased number of Transitional T3 B cells and B1a B cells in drug-resistant VL strain infection. Besides, we also observed alteration in Follicular B cell population of antimony-resistant strain infected mice. Drug-resistant strain induced secretion of elevated level of IL-10 from B1a B cells and IL-6 from Transitional T3 B cell subsets in the splenocytes. Purified splenic B cells from antimony drug-resistant strain infected mice showed decrease in the *Lyn* kinase gene expression compared to sensitive strain infected and uninfected mice. The current study provides insight into changes in host splenic B-cell subsets during experimental infection with antimony-sensitive and -resistant *L. donovani* in murine model.

## 1. Introduction

Leishmaniases, caused by the group of parasites belonging to the genus *Leishmania*, have different clinical manifestations like cutaneous, mucocutaneous and visceral forms. The visceral form (VL) of the disease, caused by *Leishmania donovani* (LD), is fatal unless treated properly. Previously antimony compounds were the mainstay of VL treatment. But the emergence of antimony-resistant LD parasites in India has compelled a shift to new drugs like miltefosine and different forms of Amphotericin B for chemotherapy [1]. Currently, 78% of the recent clinical isolates from Bihar, India are resistant to antimonials [2]. Our laboratory has been working to characterize the differences between the antimony-sensitive [SbS] and antimony-resistant [SbR]-LD parasites. We have already demonstrated that SbR-LD parasites have a different surface glycan composition from their sensitive counterpart and the Sb-resistant parasites give rise to aggressive pathology as compared to sensitive counterpart [3]. The aggressive pathology in SbR-LD infection modulates the host immune response by novel mechanisms [4]. The SbR-LD infected mice have higher spleen parasite burden compared to SbS-LD infection [5]. The resistant LD strains were found to induce a higher level of IL-10 production from macrophages, increase the expression of multi drug resistant protein [6] and trigger regulatory T cells to secrete immune-suppressing IL-10 and TGF-β [7,8]. Besides, several reports demonstrated that *Leishmania* infection stimulates immune pathology by modulating B cell biochemistry. Removal of B-cells by IgM treatment promotes resistance to cutaneous leishmaniasis [9] and B-cell deficient mice resolve LD infection more rapidly than wild type mice [10]. The B-cell repertoire in the spleen is heterogeneous in their maturation stage [11]. Bone marrow-derived immature B-cells migrate to the secondary lymphoid organ and differentiate through distinct transitional stages termed T1 and T2 before differentiating into mature follicular (FO) or marginal zone (MZ) B-cells [12,13]. Interestingly, T3 cells are a de-selected population from the developmental pathway. Thus, T1, T2, T3, FO, and MZ cells constitute splenic B2 B-cells. There is another population of mature B-cells known as B1 cells originated from the fetal liver which can be further subdivided into B1a and B1b subsets [14]. Another way to functionally divide B-cells into ‘regulatory’ and ‘effector’ B cell subsets is based on discrete cytokine-producing patterns. Regulatory B-cells are distinguished by their ability to produce IL-10 and TGF-β-1 while the ‘effector’ B-cell population produces cytokines like IL-4, IL-6 or IL-12 and TNF-α [15]. Thus, B-cells apart from immunoglobulin production can participate in immune-regulation. LD exploits endosomal Toll-like receptors (TLRs) of B-cells to induce cytokine secretion [16]. Apart from IL-10 production, IL-1, IL-6, and IFN-I are also downstream targets of endosomal TLR signaling following exposure of LD amastigotes [16]. It has been reported that increased secretion of IL-10 by B1 B cell is accountable for the increased susceptibility of host cells to VL [17]. The activity of IL-10 producing B-cells is well documented in a variety of experimental infectious diseases [18,19] and autoimmunity [20]. A member of the Src family kinase, lyn, plays a significant role in autoimmunity, where *lyn*-deficient mice show impairment of peripheral B cell proliferation [21]. Also, lyn- knock out mice produce higher level of IL-10 secreting B cells [22]. Not only the secretion of different cytokines during VL, but another intriguing feature associated with infection is the presence of autoantibodies in the sera of visceral and cutaneous leishmaniasis patients [23]. This information has been supported in murine VL, where disease exacerbation is associated with an increased level of IgM, polyclonal B cell activation, and generation of autoantibodies [24]. All this information generates considerable interest in B cells regarding their immune-modulation capacity and involvement in the pathology of VL.

In this study, we have investigated whether antimony-sensitive and -resistant LD infection modulates splenic B cells and influences secretion of disease-promoting IL-10 from any of the subsets.

## 2. Results

### 2.1. Interaction between Purified Splenic B Cells with Leishmania Promastigotes of Antimony Resistant Strains Promote Higher Level of IL-10 and IL-6 Secretion as Compared to Its Sensitive Counterpart

**Figure 1:**
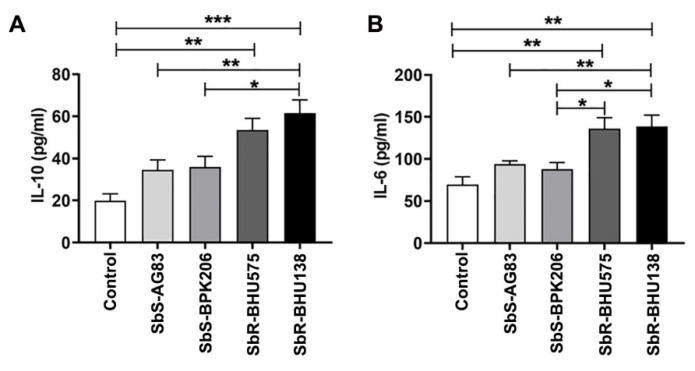
Secretion of IL-10 (**A**) and IL-6 (**B**) cytokines from purified splenic CD19+ B cells from BALB/c mice interacted with SbS-AG83, SbS-BPK206, SbR-BHU575, and SbR-BHU138 *Leishmania* strains in comparison with uninteracted control. Data were analyzed by one-way ANOVA and Tukey’s Multiple comparison test was used; level of significance are indicated by *p* values. (*n* = 5 in each group; * *p* < 0.05, ** *p* < 0.01, *** *p* < 0.001).

**Figure 2:**
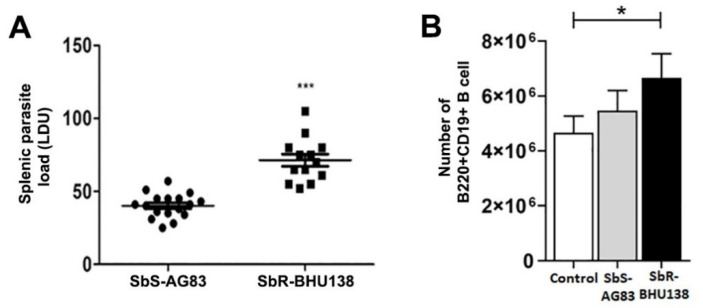
Splenic parasitic load during the second month of infection (**A**). BALB/c mice were infected with antimony-sensitive (SbS-AG83) and antimony-resistant (SbR-BHU138) *Leishmania donovani*. Data were analyzed by Mann–Whitney tests, and levels of significance are indicated by *p* values (SbS-AG83, *n* = 17; SbR-BHU138, *n* = 13; *** *p* < 0.001). The number of B220 + CD19+ B cells from the spleen of BALB/c mice upon infection with SbS-AG83 and SbR-BHU138 *Leishmania* strains in comparison with uninfected control mice (**B**). (Uninfected Control, *n* = 12; SbS-AG83, *n* = 12; SbR- BHU138, *n* = 12). Data were analyzed by one-way ANOVA and Tukey’s Multiple comparison test was used; the level of significance is indicated by *p*-values (* *p* < 0.05).

Our laboratory has earlier characterized antimony-sensitive and -resistant parasites of LD [3], where an increased level of IL-10 secretion from host immune cells plays a significant role in drug resistance mechanism. Prior reports suggested that B cells act as a source of different cytokines during *Leishmania* infection [16,25]. To identify whether drug-resistant strains induce higher level of IL-10 secretion from B cells, MACS purified CD19+ B cells (Figure S1) from spleen of uninfected control mice were cultured and incubated for 24 h with the promastigotes of four different *Leishmania* strains at the ratio of 1:10 of B cell: promastigotes. Using ELISA, we observed that B cells following interaction with promastigotes of BHU575 and BHU138, antimony-resistant strains, secreted increased level of IL -10 compared to AG83 and BPK206, antimony-susceptible strains (Figure 1A). In addition to IL-10, we also identified increased level of IL-6 in the same supernatant while interaction between B cells and promastigotes of antimony-resistant strain (Figure 1B). Further, selecting one strain each from the resistant and sensitive category, we noticed an increased splenic parasitic load in resistant strain infected mice at two months of infection (Figure 2A). In the murine model of visceral leishmaniasis, organ-specific parasitic infection prevails and a significant increase in parasitic load was reported in spleen during the second month of infection [26]. Interestingly, in the splenocytes of antimony drug-resistant SbR-BHU138 infected mice, we observed significantly increased number of B220+CD19+ B cells compared to drug-sensitive SbS-AG83 and uninfected control mice splenocytes (Figure 2B).

### 2.2. Identification of Splenic B Cell Subsets as a Source of IL-10 and IL-6 Cytokine Secretion during Leishmania Infection

**Figure 3:**
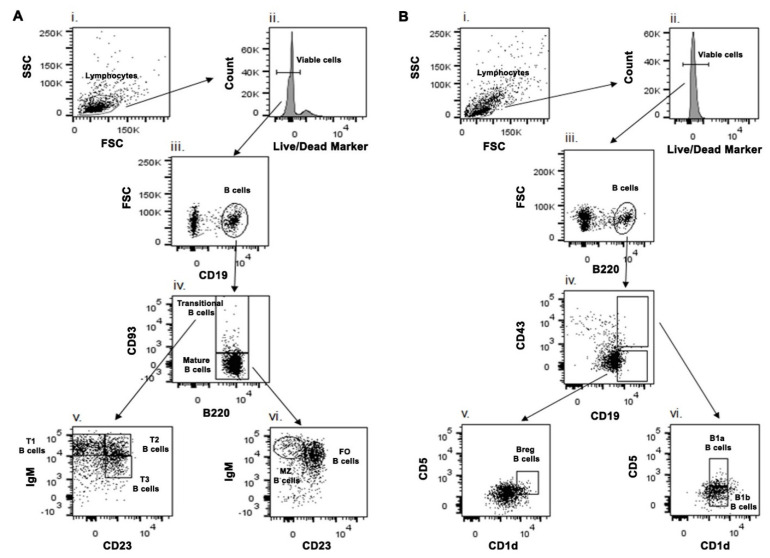
Schematic presentation of phenotypic characterization of splenic B cell subsets from BALB/c mice. (**A**) Splenic Transitional T1 (B220+CD93+IgM++CD23−), T2 (B220+CD93+IgM++CD23+), T3 (B220+CD93+IgM+CD23+), Follicular B cells (B220+CD93−CD23+IgM+/IgM++) and Marginal Zone B cells (B220+CD93−CD23−IgM++) were characterized by different cell surface markers. (**B**) Using different cell surface markers splenic B1a (CD19^hi^CD43+CD5+CD1d^mid^), B1b (CD19^hi^CD43+CD5−CD1d^mid^) and regulatory B cells (Breg) (CD19^hi^CD43−CD5+CD1d^hi^) were phenotypically characterized.

**Figure 4:**
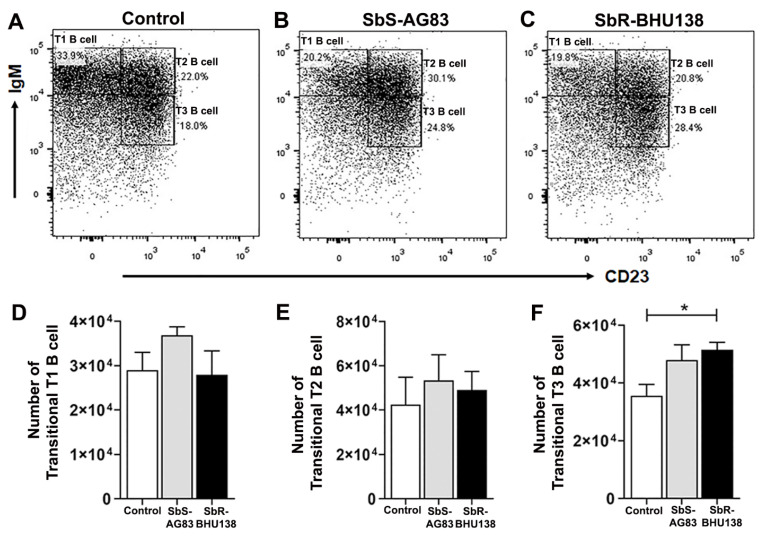
Modulation of splenic Transitional B cell subsets during infection with SbS-AG83 and SbR-BHU138 *Leishmania* strains in comparison with uninfected control mice. A representative dot plot of Transitional T1, T2 and T3 B cells from splenocytes of uninfected control BALB/c mice (**A**), mice infected with antimony-sensitive (SbS-AG83) (**B**), and antimony-resistant (SbR-BHU138) (**C**) *Leishmania donovani*. The total number of splenic Transitional T1 (**D**), T2 (**E**) and T3 (**F**) B cells during infection with *Leishmania* in BALB/c mice (Uninfected Control, *n* = 12; SbS-AG83, *n* = 10; SbR- BHU138, *n* = 12). Data were analyzed by one-way ANOVA and Tukey’s Multiple comparison test was used; the level of significance is indicated by *p* values (* *p* < 0.05).

**Figure 5:**
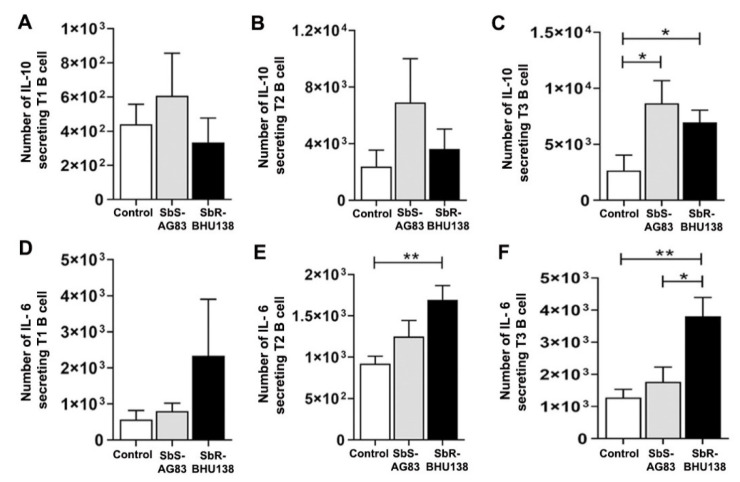
Secretion of different cytokines from Transitional B cell subsets during infection with SbS-AG83 and SbR-BHU138 *Leishmania* strains in comparison with uninfected control mice. The total number of IL-10 secreting splenic Transitional T1 (**A**), T2 (**B**)**,** and T3 (**C**) B cells during infection with *Leishmania* in BALB/c mice (*n* = 5 in each group). The total number of IL- 6 secreting splenic Transitional T1 (**D**), T2 (**E**)**,** and T3 (**F**) B cells during infection with *Leishmania* in BALB/c mice (*n* = 5 in each group). Data were analyzed by one-way ANOVA and Tukey’s Multiple comparison test was used; the level of significance is indicated by *p* values (* *p* < 0.05, ** *p* < 0.01).

To identify which specific splenic B cell subset(s) acts as a source of IL-10 and/or IL-6, we first phenotypically characterized different splenic B cell subsets from whole splenocytes (Figure 3). While characterizing Transitional B cell (B220+CD93+) subsets T1, T2 and T3 B cells (Figure 4A–C), we noticed a significant increase in the frequency (Figure S3C) as well as the number (Figure 4F) of Transitional T3 B cells (B220+CD93+IgM+CD23+) from the splenocytes of SbR-BHU138 (drug-resistant strain) infected mice compared to uninfected control mice and infection with drug-sensitive (SbS-AG83) strains. The number of splenic Transitional T1 B cell (B220+CD93+IgM++CD23−) remained unaltered between uninfected control and infected mice (Figure 4D). However, T1 B cell frequency was significantly decreased in SbR-BHU138 infected mice (Figure S3A). Further using intracellular cytokine assay, we observed a three-fold increase in the number of IL-10 secreting splenic Transitional T3 B cells while infection with both antimony drug- sensitive and -resistant strain (Figure 5C). Besides, the number of splenic IL-6 secreting Transitional T2 B cell (Figure 5E) and T3 B cell (Figure 5F) were elevated in the splenocytes of antimony-drug resistant SbR-BHU138 strain infection. Similarly, an increased frequency of splenic IL-10 and IL-6 secreting Transitional T3 B cells in resistant strain infected mice was evident (Figure S4C,F).

**Figure 6:**
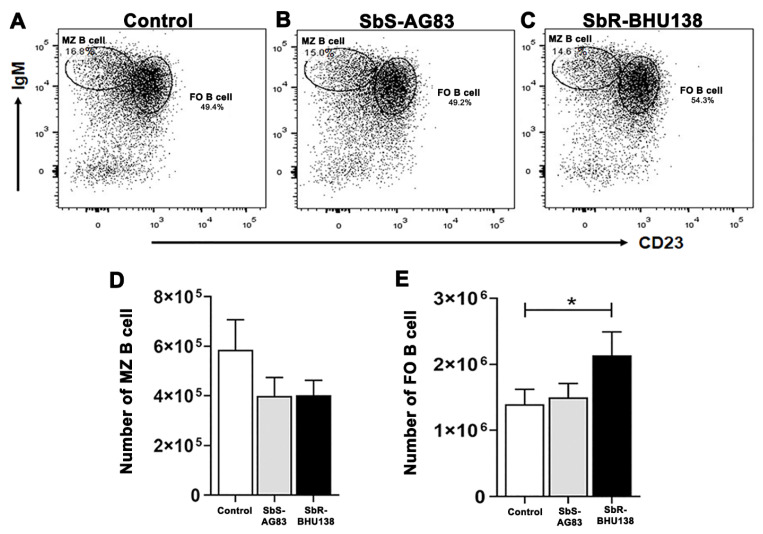
Modulation of splenic Marginal Zone (MZ) B cell and Follicular (FO) B cell subsets during infection with SbS-AG83 and SbR-BHU138 *Leishmania* strains in comparison with uninfected control mice. A representative dot plot of MZ and FO B cells from splenocytes of uninfected control BALB/c mice (**A**), mice infected with antimony-sensitive (SbS-AG83) (**B**), and antimony-resistant (SbR-BHU138) (**C**) *Leishmania donovani*. The total number of splenic MZ B cells (**D**) and FO B cells (**E**) in BALB/c mice infected with SbS-AG83 and SbR-BHU138 *Leishmania* strains in comparison with uninfected control mice. Data were analyzed by one-way ANOVA and Tukey’s Multiple comparison test was used; the level of significance is indicated by *p* values (*n* = 10 in each group; * *p* < 0.05).

**Figure 7:**
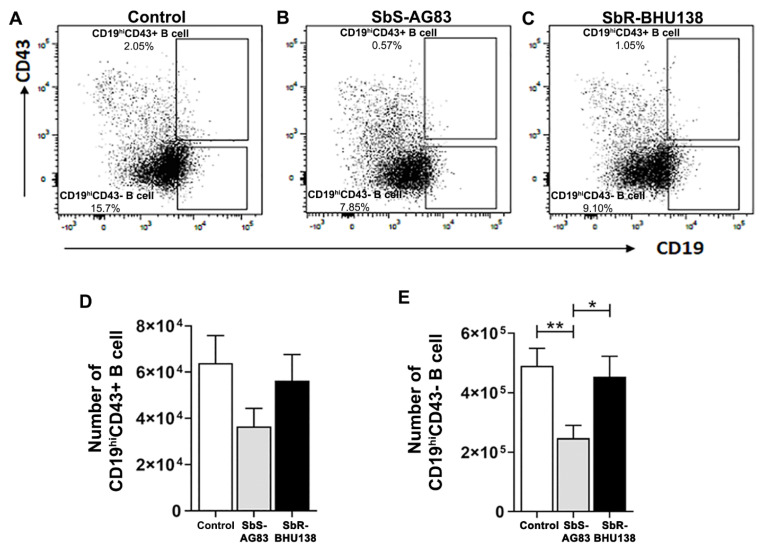
Modulation of splenic CD19^hi^CD43+ and CD19^hi^CD43− B cell subsets during infection with SbS-AG83 and SbR-BHU138 *Leishmania* strains in comparison with uninfected control mice. A representative dot plot of CD19^hi^CD43+ and CD19^hi^CD43− B cells from splenocytes of uninfected control BALB/c mice (**A**), mice infected with antimony-sensitive (SbS-AG83) (**B**), and antimony-resistant (SbR-BHU138) (**C**) *Leishmania donovani*. The total number of splenic CD19^hi^CD43+ B cells (**D**) and CD19^hi^CD43− B cells (**E**) in BALB/c mice infected with SbS-AG83 and SbR-BHU138 *Leishmania* strains in comparison with uninfected control mice. Data were analyzed by one-way ANOVA and Tukey’s Multiple comparison test was used; the level of significance is indicated by *p* values (*n* = 10 in each group; * *p* < 0.05, ** *p* < 0.01).

**Figure 8:**
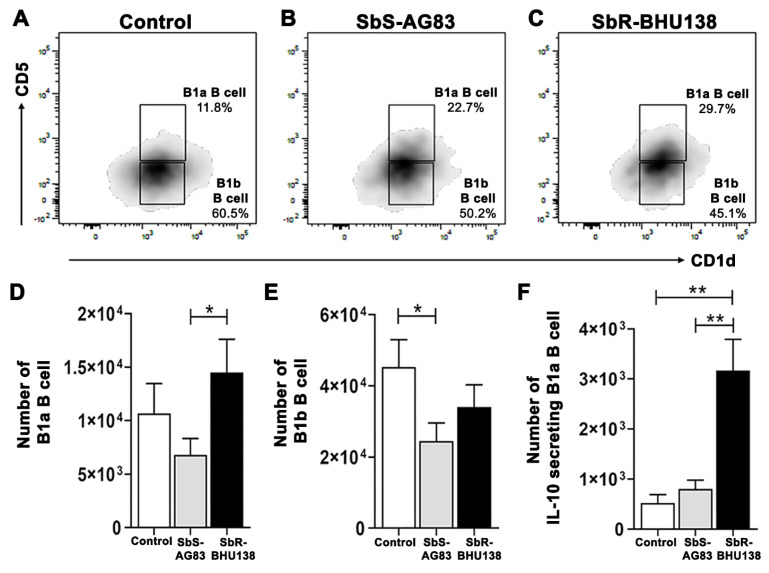
Modulation of splenic B1a and B1b B cell subsets during infection with SbS-AG83 and SbR-BHU138 *Leishmania* strains in comparison with uninfected control mice. A representative plot of B1a and B1b B cells from splenocytes of uninfected Control BALB/c mice (**A**), mice infected with antimony-sensitive (SbS-AG83) (**B**), and antimony-resistant (SbR-BHU138) (**C**) *Leishmania donovani*. The total number of splenic B1a B cells (**D**) and B1b B cells (**E**) in BALB/c mice infected with SbS-AG83 and SbR-BHU138 *Leishmania* strains in comparison with uninfected control mice (*n* = 10 in each group). The total number of IL-10 producing splenic B1a B cells (**F**) (*n* = 5 in each group) in BALB/c mice infected with SbS-AG83 and SbR-BHU138 *Leishmania* strains in comparison with uninfected control mice. Data were analyzed by one-way ANOVA and Tukey’s Multiple comparison test was used; the level of significance is indicated by *p* values (* *p* < 0.05, ** *p* < 0.01).

Next, we characterized Follicular (FO) B cell (B220+CD93−CD23+IgM+/IgM++) and Marginal Zone (MZ) B cell (B220+CD93−CD23−IgM++) (Figure 6A–C) within Mature B cell subsets and detected a significant increase in the number of splenic FO B cells in antimony-resistant *Leishmania* strain infected mice compared to uninfected mice (Figure 6E). Whereas the decrease in splenic MZ B cell numbers (Figure 6D) as well as frequency (Figure S5A), was observed in infected mice compared to uninfected control. Unlike in-vitro stimulation of B cell with *L. major* [27] we did not observe secretion of IL-10 from MZ B cells, neither secretion of IL-6 (data not shown). While stimulation with *L. major*, not only MZ like B cell, but also B1a or Breg like B cells act as a source of IL-10 [27]. Under our experimental condition, when we phenotypically characterized CD19^hi^CD43+ and CD19^hi^CD43− population (Figure 7A–C), we noticed a significant decrease in the number of splenic CD19^hi^CD43− B cells in drug-sensitive SbS-AG83 infection compared to uninfected mice as well as drug-resistant SbR-BHU138 strain infection (Figure 7E). Further characterization of splenic B1a and B1b subsets within CD19^hi^CD43+ population showed, significantly increased numbers and frequency of B1a B cells following infection with the drug-resistant strain (SbR-BHU138) compared to infection with the drug-sensitive strain (SbS-AG83) (Figure 8D and Figure S6A). In the splenocytes, a reduced number of B1b cell population was noticed in case of drug-sensitive strain infected mice (Figure 7E). Next, intracellular cytokine assay confirmed elevated level of IL-10 secreting B1a B cells in the splenocytes of drug-resistant SbR-BHU138 strain infected mice compared to drug-sensitive SbS-AG83 infected as well as uninfected control mice (Figure 7F and Figure S6B). A three-fold increase in the number as well as the frequency of IL-10 secreting B1a B cell in the splenocytes was noticed following infection with SbR-BHU138 strain compared to uninfected mice (Figure 7F and Figure S6B). While characterizing the Breg B cell population, we observed a significant reduction of their number in the splenocytes of drug-sensitive strain infected mice (Figure S7D). In our experiments, we did not observe secretion of IL-10 from Breg B cells after infection (Figure S7E).

### 2.3. Effect on Lyn Kinase While Infection with Leishmania Parasites

**Figure 9:**
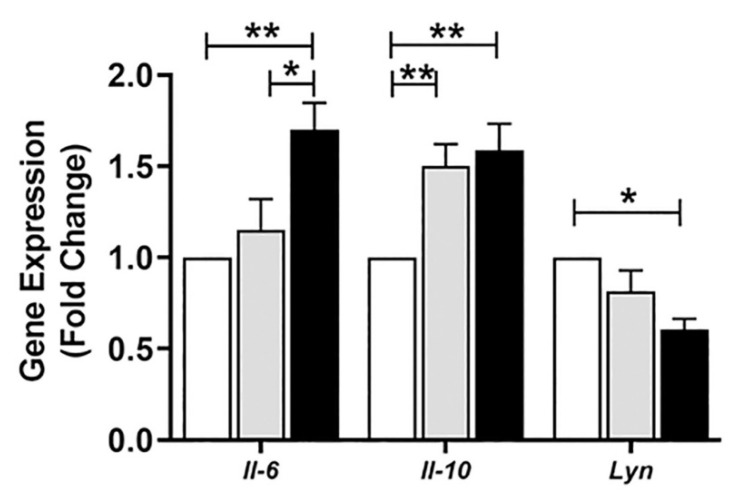
Expression of *Il-6*, *Il-10*, and *Lyn kinase* genes in the splenic B cells of antimony-sensitive (SbS-AG83) and antimony-resistant (SbR-BHU138) *Leishmania* strain infected mice in comparison with uninfected control mice. B cells were purified by MACS technique and expression studied by RT-PCR. mRNA expression levels are presented over uninfected control mice (Control = 1.0) after normalization with housekeeping gene β-actin. Data were analyzed by two-way ANOVA and Tukey’s Multiple comparison test was used; the level of significance is indicated by *p* values (Uninfected Control, *n* = 4; SbS-AG83, *n* = 4; SbR-BHU138, *n* = 4; * *p* < 0.05, ** *p* < 0.01).

After observing the changes in the population of different splenic B cell subsets during infection with antimony drug-sensitive and -resistant *Leishmania* strains, we wanted to identify the host B cell factor affected by the parasite. In our experimental condition, we observed a significant proliferation of splenic B1a B cells, whereas the number and frequency of MZ B cells decreased in antimony drug-resistant infected animals. In the differentiation and development of B cell, tyrosine-protein kinase Lyn, plays a significant role [28]. Lyn kinase knock-out mice develop severe splenomegaly along with the increased secretion of IL-10 [22]. There are reports that Src family kinases Hck, Lyn and Fgr facilitate IgG- mediated phagocytosis and *Leishmania* infection [29]. Since we observed B-cell subsets being affected, we investigated the mRNA level of Lyn tyrosine kinase in MACS purified B cell from the splenocytes of infected mice. The mRNA level of Lyn kinase was downregulated in the B cell of antimony drug-resistant LD infected mice in comparison to its antimony drug-sensitive counterpart and uninfected mice, respectively (Figure 9). Besides, we also noticed upregulation of IL-6 and IL-10 in the B cell from infected mice (Figure 9).

## 3. Discussion

To date, there is not enough information regarding alteration of splenic B cell subsets and the generation of disease-promoting cytokines during the pathogenesis of antimony-resistant visceral leishmaniasis. In our present work, the ex-vivo study of cytokine secretion shows increased level of IL-10 and IL-6 from MACS purified splenic CD19+ B cells following interaction with antimony drug-resistant SbR-LD (BHU138) promastigotes compared to drug-sensitive SbS-LD (AG83) promastigotes (Figure 1). This indicates that VL infection could influence B cell differentiation and development. In continuation, we noticed infection-induced splenomegaly along with an increase in the number of B220+CD19+ B cells in the splenocytes of antimony drug-resistant strain infected mice (Figure 2). However, the frequency of B220+CD19+ B cells did not alter significantly (Figure S2). Next, we phenotypically characterized different splenic B cell subsets [11,30] to identify the B cells secreting IL-10 and IL-6. In the splenic Transitional B cell subsets, we noticed increased frequency as well as the number of Transitional T3 B cells in the splenocytes of SbR-LD infected mice (Figure 4F and Figure S3C). Further, we noticed an increase in the number of FO B cells in the splenocytes of drug-resistant SbR-BHU138 strain infected mice (Figure 6E). Besides, the number of MZ B cells decreased following infection (Figure 6D). This information point towards the dichotomy of resistant and sensitive strain infection in the host B cell differentiation and development. In case of infection with *L. infantum* in BALB/c mice, it was reported that during eight weeks of infection there was an increase in CD19+CD23+ B cells [31]. In that study, Perez-Cabezas et al. also noticed an increase in CD4+CXCR5+PD1+ T cells during infection [31]. Similarly, in our present work, we noticed an increase in CD23+ Transitional T3 B cell population. The expression of CD23 on B cell contributes to the developmental process from immature to transitional leading to mature B cell [32]. CD23+ Transitional B cells are involved in the crosstalk with T cells [32]. This productive interaction between B and T cells is necessary to counter foreign antigens during infection [33]. Also, this interaction plays a role to maintain a normal immune repertoire and safeguards from autoimmunity [32,34]. Of note, chronic infection in mice with VL, MZ region of spleen is affected leading to loss of Marginal zone Macrophages [35]. In the present study, the alteration of FO and MZ B cell homeostasis during infection might indicate a loss of protective anti-parasite responses in *Leishmania* infection. This dysregulation and variation of protective immune response could be one of the reasons behind the failure of vaccine development during leishmaniasis. The decreased number of CD19^hi^CD43+ and CD19^hi^CD43− B cell populations in the splenocytes of drug-sensitive strain infected mice added further complexity in this dichotomy (Figure 8D,E). While studying B1a B cell subsets in the splenocytes of infected mice, we noticed a significant increase in the frequency as well as the number of B1a B cell population after infection with SbR-LD strain compared to SbS-LD strain (Figure 8D and Figure S6A). We have also observed a significant decrease in the number of regulatory B cells in the splenocytes of drug-sensitive strain infected mice (Figure S7D). This dichotomy in splenic B cell differentiation during infection with antimony-sensitive and resistance strains will further help to understand the modulation of host immune system during active infection. Studies with purified spleen B cells and comparative study with various sensitive and drug-adapted laboratory resistant strains infected mice may add further knowledge in this regard. It is well known that infection, cytokine secretion, and inflammation affects lymphopoiesis [36], which could be the reason for variability in the peripheral B cell differentiation and development during drug-sensitive and -resistant strain infection.

Interestingly, cytokine plays a major role in the dichotomy of drug-sensitive and -resistance behavior in VL [37]. In our studies, we observed increased frequency as well as the number of IL-10 secreting Transitional T3 B cells from infected animals (Figure 5C and Figure S4C). In addition to IL-10 secreting T3 B cells, frequencies, as well as the number of IL-10 secreting B1a B cells, were significantly higher in SbR-LD strain compared to its sensitive counterpart (Figure 8F and Figure S6B). While in-vitro stimulation with *L. major*, the IL-10 producing B cells were reported as Transitional T2 like and MZ like B cells [27]. The increased secretion of IL-10 might influence host cell susceptibility towards infection and masking of an immune response. It was reported that secretion of IL-10 by B1 B cell-dependent manner during VL impairs peritoneal macrophage’s protective role in managing pathogenesis [17]. Similarly, in the experimental mice model with *L. chagasi* infection, IL-10 secretion by B1 B cells precedes host susceptibility while infection with the parasite [38]. This adds to the knowledge of differential immune regulation during heterogeneous pathogen–host interaction in *Leishmania* infection. The heterogeneous immune response has been reported during infection with *L. chagasi*, where differential effects on B and T cells were noticed in three different experimental inbred mouse strain [38]. However, in human Chagas disease, B1 B cells play opposite role, where CD11b+ B1 B cells are responsible for protective immune response [39]. The interaction between *L. donovani* amastigotes and purified splenic B cell induces IL-10 secretion by MZ B cell and Breg B cells leading to disease exacerbation [40]. Purified human tonsillar B cell, when exposed to *Leishmania infantum* parasites, stimulates IL-10 secretion from CD19+CD24+CD27− cell populations, which function as regulatory B cells [41]. These findings highlight the involvement of regulatory B cell as source of IL-10 in disease pathogenesis. However, in our experimental condition, we did not observe Breg B cell as a source of secretion of IL-10 (Figure S7E). Besides, we noticed an increased level of IL-6 secreting Transitional T2 (Figure 5E) and T3 B cells (Figure 5F) in SbR-LD strain infected mice. Earlier it was reported that IL-6 deficient mice showed enhanced protection with antimony therapy against VL [42]. In our experimental condition, we observed Transitional T3 B cells acting as a source of both IL-10 and IL-6 cytokines. There is a report that purified peritoneal B1 B cells express IL-10, IL-6, and TNF-α, when stimulated with extracellular vesicles released by *Leishmania amazonensis* [43]. In the active diseased stage, VL patient shows an elevated level of serum IL-10, IFN-γ, and IL-6 [44], which suggests the involvement of IL-6 in disease pathology. Ability of drug-resistant *Leishmania* parasites to promote aggressive pathology is linked with their ability to influence both macrophages and T cells to produce more IL-10. This study shows that increased virulence during infection with Sb-resistant *Leishmania* parasites may also be partly due to their ability to differentially influence the B cells compared to the Sb-sensitive counterpart. Also, in experimental autoimmune encephalomyelitis (EAE), MZ B cells and Transitional B cells act as a source of both IL-6 and IL-10 cytokines [45]. The generation of different types of regulatory cytokines during infection may be because of various reasons. It may be because the kinetics of stimulation varies in vivo which in turn is dependent on the dose and signals or a combinatorial quality [45].

As we observed changes in different splenic B cell subsets during infection, we suspected that presence of parasites in the spleen could influence B cell development and differentiation. Interestingly, we observed the downregulation of the *Lyn kinase* gene in purified splenic B cells from SbR-LD infected mice compared to its sensitive counterpart and uninfected control (Figure 9). Loss of Lyn kinase affects B cells and generates autoimmune diseases [21]. Lyn-deficient mice have shown decreased splenic Transitional T1, T2, and T3 B cell numbers in comparison to wild-type animals [28]. Marginal Zone B cell was affected in knocked out mice and significant proliferation of spleen B1 B cell were noticed. These mutant mice develop ribonucleoprotein and anti-DNA autoantibodies. Also, Lyn^-/-^ mice developed splenomegaly and showed expansion of different B cell subsets [22]. The IL-10 secreting B cells from Lyn knock-out animals are plasma cells/plasmablasts, Transitional T1 and T2, and B1a/b cells. Increased level of IL-6 was reported from knock-out mice [22,28]. Not only B cell subsets, the population of myeloid cells, monocytes/macrophages, and granulocytes also increased while B cell-specific deletion of Lyn kinase in mouse model [28]. The production of IL-10 by B cells from Lyn knock-out animals could be to reduce inflammatory response caused by myeloid cell activation. Like Lyn knock-out animals, presence of ribonucleoprotein has been reported in the sera of visceral leishmaniasis patients [23]. In our experimental condition, we noticed a decrease in *Lyn kinase* expression in the B cell of SbR-LD infected mice, which could activate the inflammatory component in the cell. The secretion of IL-6 from B cells may induce the development of other IL-10 secreting B cells or other immune cells. To circumvent the inflammatory effect, B cells generate IL-10, which could also induce other immune cells, such as regulatory T cells to produce IL-10.

It remains difficult to determine whether secretion of different cytokines by host cells is directly regulated by parasitic infection or they are the actions of host immunity to control infection. This study lays the foundation for understanding the complexities of B cell differentiation and function during antimony-sensitive and -resistant *Leishmania* *donovani* infection.

## 4. Materials and Methods

### 4.1. Parasites

For our experimental purpose, we used four different strains of *L. donovani* from Indian subcontinent, characterized based on their sensitivity towards sodium antimony gluconate (SAG) drug [3]. Pentavalent antimonial-sensitive strains (SbS-LD) were AG83 (MHOM/IN/83/AG83) and BPK206 (MHOM/NP/03/BPK206/0), whereas BHU575 (MHOM/IN/09/BHU575/0) and BHU138 (MHOM/IN/05/BHU138) were antimonial-resistant strains (SbR-LD). Promastigotes were grown in medium M199 (Invitrogen, Carlsbad, CA, USA) supplemented with Hanks’ salt containing HEPES (12 mM), L-glutamine (20 mM), 10% heat-inactivated fetal bovine serum (FBS), 100 U/mL penicillin, and 100 μg/mL streptomycin (Invitrogen). All these strains are clinical isolates from the hyperendemic zone of Bihar state in India and were characterized earlier from our laboratory by in-vitro studies. The sensitive and resistance strains were classified based on half maximal effective concentration (EC_50_) of antimonial [3]. In that study our group showed that resistant strains have higher expression of thiol metabolizing enzyme, upregulation of ATP binding cassette transporter multidrug resistance protein 1, and permeability glycoprotein. SbR strains showed higher level of N-acetyl-D-galactosaminyl residue on the surface of promastigote membrane in comparison with SbS strains [3]. Higher level of IL-10 secretion from infected macrophages was correlated with SbR strains. Our group also showed that SbR parasites lacking this glycan on the surface behave like SbS parasites [6].

### 4.2. Determination of Splenic Parasitic Load

Two different strains (AG83 and BHU138) of *L. donovani* were used to infect female BALB/c mice (4 to 6 weeks old). Parasites were maintained by passage in Syrian golden hamsters. Mice were infected i.v. via the lateral tail vein with 1 × 10^7^ promastigotes in 200 µL of PBS. The course of visceral infection was determined by examining methanol-fixed, Giemsa-stained imprints of the cut spleen, and quantified organ parasite burden as Leishman–Donovan units (LDU) using the formula: LDU = [(number of parasites/1000 host nuclei) × organ weight in milligrams (mg)] [10]. Earlier, our laboratory has characterized parasitic burden by both LDU and limiting dilution method using the above-mentioned parasites [6,46]; in the present study parasitic burden was checked by LDU.

### 4.3. Reagents and Chemicals

The following antibodies were used for staining: anti-mouse IgM-APC, B220-FITC, CD93-PE CF594, CD19-Alexa Fluor 647, IL10-PE, CD23-BB515, CD5-PerCP, CD1d-BV421, CD43-PE Cy7, B220-APC Cy7, Fc Blocker (CD16/CD32), Cytofix/CytoPerm kit (all from BD Pharmingen), CD23-PerCP/Cy5.5, CD93-PE Cy7, CD1d-PB, CD5-PE Cy7, CD21-APC Cy7, CD19-PE, CD43-APC Cy7 (all from Bio Legend), Live/Dead aqua marker (Molecular Probes).

### 4.4. Phenotypic Characterization of B Cell Subsets Using Different Cell Surface Markers

Splenocytes were isolated from uninfected and infected mice and cultured in RPMI 1640 medium containing 10% heat-inactivated fetal bovine serum (FBS), 100 U/mL penicillin and 100 μg/mL streptomycin (Invitrogen) for 24 h. Before staining for surface markers, cells were incubated with an Fc blocker for 30 min to minimize nonspecific staining. Cells were stained with relevant antibodies on ice for 30 min in PBS buffer containing 2% FBS, then washed twice with PBS buffer containing 2% FBS before data acquisition by BD FACS ARIA II flow cytometer. Analysis was performed using FlowJo software (Tree Star). Live cells were gated based on forward and side scatter profiles and based on exclusion with live/dead aqua marker. Then the cells were gated with CD19. These CD19+ B cells were further gated with B220 and CD93 to characterize Transitional B cell (B220+CD93+) and mature B cell (B220+CD93−) [30]. Transitional B cells were further gated with IgM and CD23 for characterizing T1, T2 and T3 Transitional B cells. Similarly, using IgM and CD23, Mature B cells were further characterized as MZ and FO B cell (Figure 3A). For characterizing B1 B cell and regulatory B cell subsets, gating strategy according to Baumgarth’s (2011) [11] was adopted (Figure 3B). Live cells were gated by B220 surface marker. These B220+ B cells were further gated with CD19 and CD43. The CD19^hi^CD43+ B cells were then gated with CD5 and CD1d to characterize B1a (CD5+CD1d^mid^) and B1b (CD5−CD1d^mid^) B cell subsets. Further, CD19^hi^CD43− B cells were gated with CD5 and CD1d to characterize Breg B cells (CD5+CD1d^hi^). Gates for positive controls were used with beads stained with individual fluorochrome and negative control taken as unstained samples in the method set up.

### 4.5. Intracellular Staining of Cytokines

For intracellular detection of IL10 and IL6 from splenic B cell subsets, splenocytes were isolated from uninfected and infected mice and cultured in RPMI 1640 medium containing 10% heat-inactivated fetal bovine serum (FBS), 100 U/mL penicillin and 100 μg/mL streptomycin (Invitrogen) for 24 h with the last 4 h with Brefeldin A. Cells were surface stained as per above-mentioned protocol. Then cells were permeabilized and fixed for 20 min on ice. After washing, cells were stained with fluorochrome tagged IL-10 and IL-6 antibody, washed, and analyzed by flow cytometry analysis. Data was acquired on BD FACS ARIA II.

### 4.6. Purification of B Cells and Analysis of Criteria of Purity

Splenic B cells from BALB/c mice were isolated and macerated over 100 µM cell strainers in ice cold PBS (pH 7.4). Then residual erythrocytes were lysed with hypotonic buffer, splenocytes were washed, counted, and purified through Magnetic Associated Cell Sorter purified (MACS) with B cell isolation kit (Stem Cell Tech. Incs, Vancouver, BC, Canada) using MACS column followed according to the mentioned protocol. These negatively selected MACS purified CD19+ B cell was further confirmed with PE- CD19 fluorochrome marker through Fluorescence Associated Cell Sorter (FACS) analysis.

### 4.7. RT-PCR Analysis

B cells from infected and non-infected mice were MACS purified using B cell isolation kit (Stem Cell Tech. Incs). After extraction of total RNA, cDNA was synthesized using SuperScript III First strand synthesis system kit (Invitrogen, Cat No. 18080051). The quantitative RT-PCR was performed for *Il-6*, *Il-10*, and *Lyn kinase* genes by SYBER Green method, and the results were analyzed by the comparative threshold cycle method and normalized by β-actin as an internal control. The PCR amplification was performed using following primers: Il-6 For: 5′-GAC AAA GCC AGA GTC CTT CAG AGA G-3′, Il-6 Rev: 5′-CTA GGT TTG CCG AGT AGA TCT C-3′; Il-10 For: 5′- CCA GTT TTA CCT GGT AGA AGT GAT G-3′, Il-10 Rev: 5′- TGT CTA GGT CCT GGA GTC CAG CAG ACT C-3′; Lyn For: 5′-CAT AGC CTG AGT TAG TTC CCT AGC-3′, Lyn Rev: 5′-TCA CAT ATG AAC ATG TGT GTA CAT GTC-3′; β-actin For: 5′-TAC CAC TGG CAT CGT GAT GGA CT-3′, β-actin For: 5′-TTT CTG CAT CCT GTC GGC AAT-3′.

### 4.8. Statistical Analysis

Data presented as mean values ± SEMs. All graphs and statistical analyses were performed using Graph pad prism 5.01 (Graph Pad Software, San Diego, California, USA) and data were analyzed by one-way ANOVA and Tukey’s multiple comparison test. *p* values less than 0.05 are considered as significant.

## Data Availability

Data is contained within the article and Supplementary Material.

## Supplementary Materials

The following are available online at https://www.mdpi.com/article/10.3390/pathogens10070814/s1, Supplementary information of Materials and Methods. Figure S1: B-cell purification by Magnetic Associated Cell Sorter (MACS), Figure S2: Percentages of B220+CD19+ B cells from spleen of BALB/c mice upon infection with SbS-AG83 and SbR-BHU138 *Leishmania* strains in comparison with uninfected control mice, Figure S3: Modulation of splenic Transitional B cell subsets in BALB/c mice while infection with SbS-AG83 and SbR-BHU138 *Leishmania* strains in comparison with uninfected control mice, Figure S4: Secretion of different cytokines from splenic Transitional B cell subsets in BALB/c mice while infection with SbS-AG83 and SbR-BHU138 *Leishmania* strains in comparison with uninfected control mice, Figure S5: Modulation of splenic Marginal Zone and Follicular B cells while infection with antimony drug-sensitive and -resistant *Leishmania* strains, Figure S6: Modulation of splenic B1a B cells while infection with antimony drug-sensitive and -resistant *Leishmania* strains, Figure S7: Modulation of splenic Breg B cells while infection with antimony drug-sensitive and -resistant *Leishmania* strains.
