# Supplementary material for: Modulation of Splenic B Cell Subsets during Experimental Leishmania donovani Infection in BALB/c Mice
Source: Pathogens. 2021 Jun 29;10(7):814. doi: 10.3390/pathogens10070814 (PMC8308600; doi:10.3390/pathogens10070814)
Supplement: Supplementary file 1 [file pathogens-10-00814-s001.zip › pathogens-1260834-supplementary.pdf]

Supporting Materials

Modulation of Splenic B Cell Subsets during Experimental *Leishmania donovani* Infection in BALB/c Mice

Koushik Mondal [1,3,*], Shantanabha Das [2], Khudiram Naskar [1] and Syamal Roy [1]

[1] CSIR-Indian Institute of Chemical Biology, Kolkata, India; drsyamalroy@yahoo.com (S.R.); knaskar08@gmail.com (K.N.); mkoushik9@gmail.com (K.M.)
[2] Department of Zoology, Diamond Harbour Women's University, Sarisha, South 24 Parganas, India; shantanabha2008@gmail.com (S.D.)
[3] The University of Tennessee Health Science Center, Memphis, USA; kmondal@uthsc.edu (K.M.)
* Correspondence: kmondal@uthsc.edu

Supporting Information

Supplementary Figures

Figure S1:

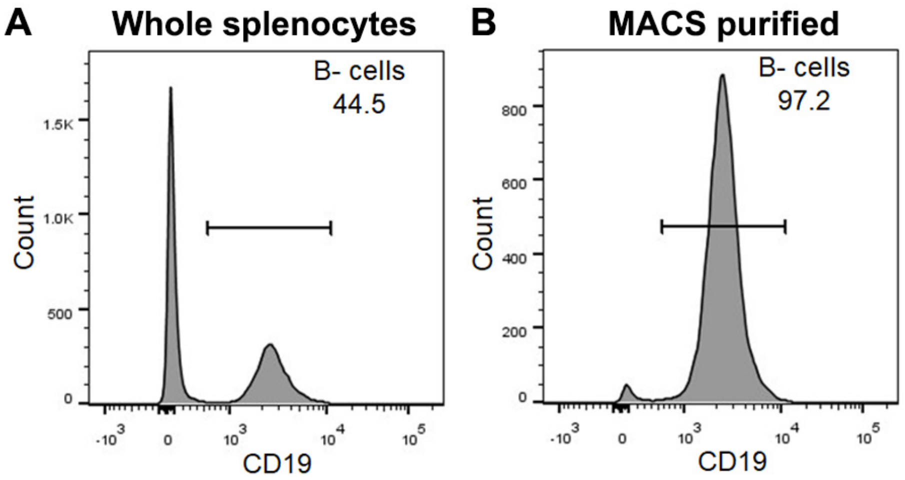

Figure S1: B-cell purification by Magnetic associated cell sorter (MACS). (A) Before purification with MACS (whole splenocytes). (B) After purification with MACS.

Figure S2:

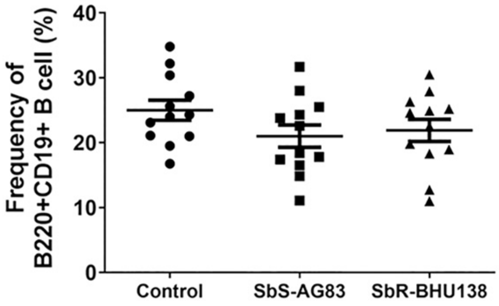

Figure S2: Percentages of B220+CD19+ B cells from spleen of BALB/c mice upon infection with SbS-AG83 and SbR-BHU138 *Leishmania* strains in comparison with uninfected control mice. Data were analyzed by one-way ANOVA and Tukey's Multiple comparison test shows no significance difference between control and infection (Control, n=12; SbS-AG83, n= 12; SbR-BHU138, n=12).

Figure S3:

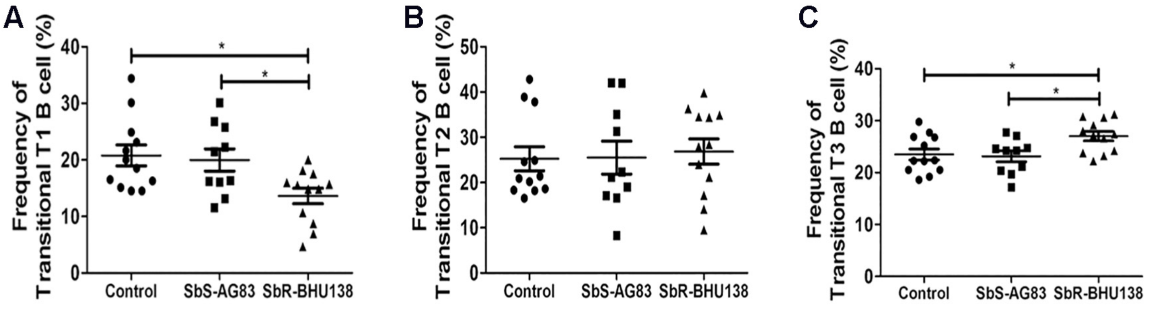

Figure S3: Modulation of splenic Transitional B cell subsets in BALB/c mice while infection with SbS-AG83 and SbR-BHU138 *Leishmania* strains in comparison with uninfected control mice. Frequency of splenic Transitional T1 **(A)**, T2 **(B)** and T3 **(C)** B cells while infection with *Leishmania* in BALB/c mice (Control, n=12; SbS-AG83, n= 10; SbR-BHU138, n=12). Data were analyzed by one-way ANOVA and Tukey's Multiple comparison test was used; the level of significance is indicated by p values (*p < 0.05).

Figure S4:

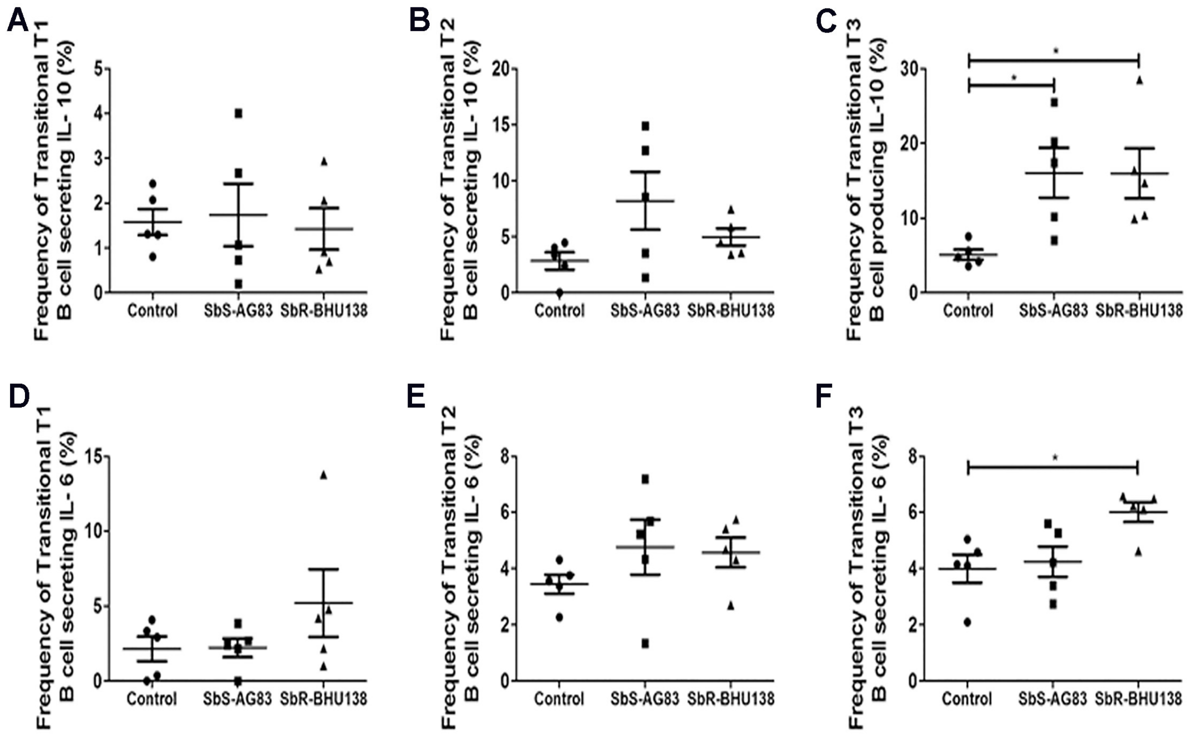

Figure S4: Secretion of different cytokines from splenic Transitional B cell subsets in BALB/c mice while infection with SbS-AG83 and SbR-BHU138 *Leishmania* strains in comparison with uninfected control mice. Frequency of IL-10 secreting splenic Transitional T1 **(A)**, T2 **(B)** and T3 **(C)** B cells while infection with *Leishmania* in BALB/c mice (n=5 in each group). Frequency of IL- 6 secreting splenic Transitional T1 **(D)**, T2 **(E)** and T3 **(F)** B cells while infection with *Leishmania* in BALB/c mice (n=5 in each group). Data were analyzed by one-way ANOVA and Tukey's Multiple comparison test was used; the level of significance is indicated by p values (*$p < 0.05$).

Figure S5:

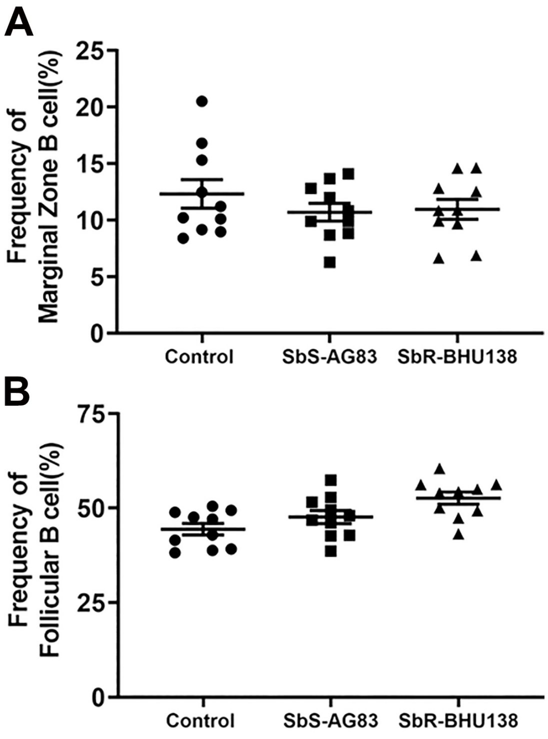

Figure S5: Modulation of splenic Marginal Zone and Follicular B cells while infection with antimony drug-sensitive and -resistant *Leishmania* strains. Frequencies of splenic Marginal Zone B cells **(A)** and Follicular B cells **(B)** in BALB/c mice infected with SbS-AG83 and SbR-BHU138 *Leishmania* strains in comparison with uninfected control mice. Data were analyzed by one-way ANOVA and Tukey's Multiple comparison test shows no significance difference between control and infection (n=10 in each group).

Figure S6:

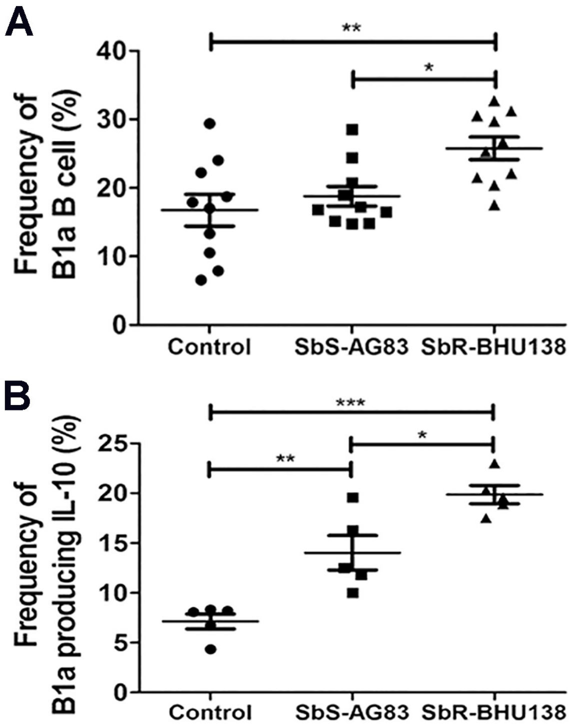

Figure S6: Modulation of splenic B1a B cells while infection with antimony drug-sensitive and -resistant *Leishmania* strains. Frequencies of splenic B1a B cells (n=10 in each group) **(A)** and IL-10 producing B1a B cells (n=5 in each group) **(B)** in BALB/c mice infected with SbS-AG83 and SbR-BHU138 *Leishmania* strains in comparison with uninfected control mice. Data were analyzed by one-way ANOVA and Tukey's Multiple comparison test was used; the level of significance is indicated by p values (*$p < 0.05$, **$p < 0.01$, ***$p < 0.001$).

Figure S7:

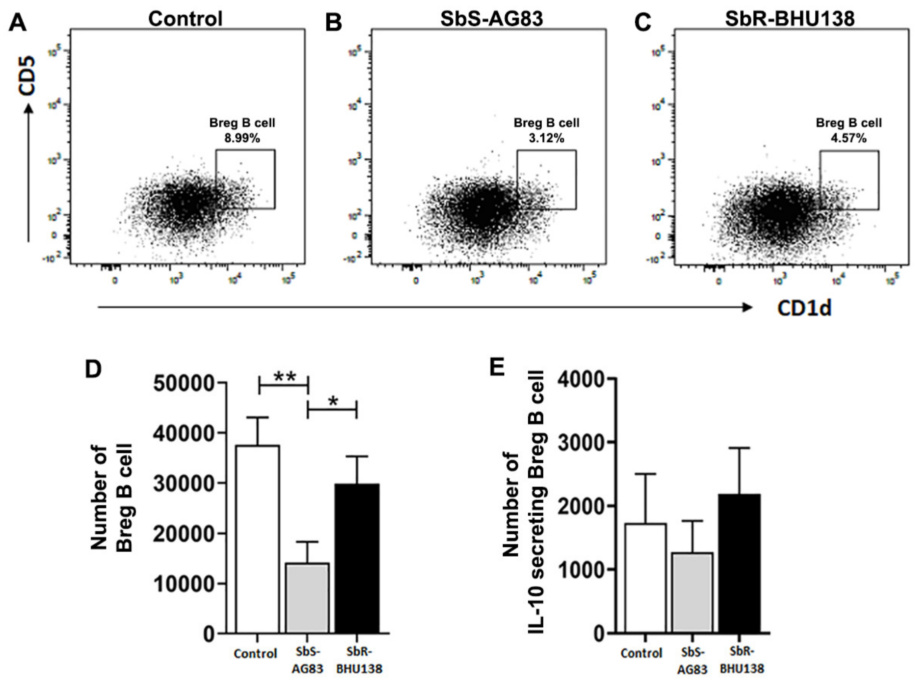

Figure S7: Modulation of splenic Breg B cells while infection with antimony drug-sensitive and -resistant *Leishmania* strains. A representative dot plot of Breg B cells from splenocytes of uninfected control BALB/c mice (**A**), mice infected with antimony-sensitive (SbS-AG83) (**B**) and antimony-resistant (SbR-BHU138) (**C**) *Leishmania donovani*. Number of splenic Breg B cells (n=10 in each group) **(D)** and IL-10 producing Breg B cells (n=5 in each group) **(E)** in BALB/c mice infected with SbS-AG83 and SbR-BHU138 *Leishmania* strains in comparison with uninfected control mice. Data were analyzed by one-way ANOVA and Tukey's Multiple comparison test was used; the level of significance is indicated by p values (*$p < 0.05$, **$p < 0.01$).
